# A United Policy in the Coming Elections

**Published:** 1921-01-29

**Authors:** 


### A UNITED POLICY IN THE COMING ELECTIONS.

### The London Centre's Meeting.

INTEREST and animation were the prevailing notes at the meeting of the London Centre on January 25, held in the beautiful Court Room of Guy's Hospital, to consider the policy of the Centre with reference to the forthcoming elections to the Council of the College. Nurses are finding their voices in public meeting, and the fertility in ideas which was evinced at this meeting must be highly encouraging to members, and to all those responsible for the management of this and other Centres, whose activity and energy could not fail to produce this awakening.

Miss Amy Hughes, in the regrettable absence of Miss Gibson, was in the chair, and was supported on the platform by members of the Executive Committee of the Centre. There was an excellent attendance, and amongst those present were Miss Alsop (Kensington Infirmary), Miss Montgomery (the Middlesex Hospital), Miss Jolley (Prison Nursing Service), Miss Barton (Chelsea Infirmary), Miss Rosalind Paget (the Midwives' Institute), Miss Redl (Brompton Hospital), and Miss Rundle.

Miss Hughes, in stating the object of the meeting, remarked that it rested with the members of the College, and more particularly of the Centres, to have a proper, good, and proportionate representation on the Council; that the voting at the election would be, as it has always been, absolutely confidential, but that such meetings should be helpful in enabling the members to agree who are the best candidates to be nominated. The aim is to have a larger number of members on the Council representative of different interests and different areas, for the feeling exists that a number of nurses are not represented properly.

The honorary officers then presented their reports, which we hope to deal with next week.

THE NOMINATION OF CANDIDATES.

The great business of the meeting was then proceeded with. Miss Biggar opened the discussion on the coming election, and referred to her letter, which has been circulated to all the Centres, dealing with area and professional representation. She stated, however, these are not the chief qualifications in a member; much more important is it to secure candidates who will uphold the policy of the College,

(*Continued on p. viii.*)

and who, while bringing experience, wide and well-balanced views, are open to new ideas. She read letters from the following Centres in relation to the forthcoming elections: Cornwall, Yorkshire Centres at Leeds, Northumberland, and Durham. Also a letter from Miss Thicknesse, late assistant matron at Crumpsall Infirmary, who considers there are too many medical men on the College Council, and not enough Poor-law representation; she suggested the matron of Crumpsall Infirmary (Miss Burgess) as an excellent candidate.

In the discussion which followed, Miss Todd (matron of St. James's Infirmary, Balham), Miss Redl (Brompton Hospital), Miss Cowlin, Miss Barton (Chelsea Infirmary), Miss Dalton (Victoria Park Hospital), Miss Nicholls (Maternity Section Royal Free Hospital), and others took part. The majority urged the claims of the small and special hospitals to be represented on the Council; their difficulties, it was stated, were great, more especially since the age for training in general hospitals has been reduced to twenty-one; a plea was put in for greater representation of the Poor-law infirmaries; and Miss Todd desired to see the constitution of the College altered so that it should provide for a stated representation of the various interests, classes, and areas. Miss Cowlin thought that enough had been said on behalf of the working nurse, and that until they were financially strong enough to make it worth the while of some working nurse to give up the necessary time to attend the meetings of the Council it would be better to talk less about her, and to aim at the representation of her interests through co-operation with the matron. Miss Nicholls thought much may be done to elect the right member if the matrons of the large hospitals would select a suitable sister, or a qualified nurse leaving to take up private nursing, and see to it that she gets sufficient votes to return her as a Council member. The claims of nurse-masseuses to representation were also put forward by another speaker, and it was pointed out that Miss Hogg, the matron of Guy's Hospital, who is already a member of the Council, may be considered to represent them.

Eventually the following names were put forward for nomination: Lady Cowdray (by Miss Cox-Davies); Miss Edmunds, sister-tutor at St. James's Infirmary, Balham, and at present a member of the Executive of the London Centre, to represent small hospitals and infirmaries (by Miss Todd); Miss J. W. Macdonald, a nurse with experience of children who is at present taking a course of training in welfare work at the London School of Economics, and is settling in London (by Miss Sheldon, Guy's Nurses' Institute); Miss Alsop (by Miss Barton); Miss Masters, matron Leicester Poor-law Infirmary, to represent the provinces and Poor-law (by Miss Cowlin); Miss Sordy, matron Queen Mary's Hospital; Miss Spackman, matron National Hospital, Queen Square; Miss Redl, matron Brompton Hospital, to represent special hospitals; Miss Cox-Davies, Royal Free Hospital, and Miss Amy Hughes (the two latter are retiring members of the Council).

It was suggested that such of these ladies as are willing should be invited to attend a meeting of the Centre, to be held in February, to give each a short address, and thus to enable the members to decide whom to put forward as official candidates. Obviously some weeding out is necessary, as with only eight vacancies for England and Wales, and nineteen Local Centres (excluding Scotland), each Centre must limit the number of its official candidates. Stress was laid on the point in the course of the meeting that no one should accept nomination who is unable to spare the time to attend the meetings of the Council. It was also urged that it would be an excellent thing to have on the Council persons who are neither doctors nor nurses. The unbiased opinions of "outsiders" would be invaluable.

